# Causal relationship between gut microbiota and viral infectious disease: A 2-sample Mendelian randomization study

**DOI:** 10.1097/MD.0000000000043258

**Published:** 2025-07-04

**Authors:** Hao Hu, Feng Xie, Sixia Jiang, Ya Song, Junjun He, Sijie Zhu, Shirui Yu, Xudong Liu

**Affiliations:** a School of Food Engineering, Moutai Institute, Renhuai, Guizhou Province, China; b Guizhou Health Wine Brewing Technology Engineering Research Center, Moutai Institute, Renhuai, Guizhou Province, China.

**Keywords:** gut microbiota, inverse variance weighting, Mendelian randomization, viral infectious diseases

## Abstract

The interaction of the dysbiosis of the gut microbiota (GM) with viral infectious disease (VID) has drawn our attention, and a better understanding of the effects of GM on VID might provide potential therapeutic approaches. However, the causal effects between GM and different types of VID remain unclear. We aimed to reveal the causal relationships between GM and VID, including adenovirus, cytomegalovirus, Epstein-Barr virus (EBV), influenza virus subtype H1N1 (H1N1) and herpes simplex virus 1 (HSV-1) infection. The GM and 5 VID types, namely, adenovirus, cytomegalovirus, EBV, H1N1 and HSV-1, were identified from large-scale genome-wide association study summary data. We used Mendelian randomization (MR) to investigate the causal relationships between GM and the 5 types of VID. MR-Egger, weighted median, inverse variance weighted, simple mode and weighted mode were used to examine the causal associations between GM and VID. Inverse variance weighting was used as the main statistical method. Sensitivity analysis was performed via the MR-Egger regression intercept method and Cochran’s *Q* test. There were 9 positive and 14 negative causal effects between genetic liability in the GM and 5 types of VID. Specifically, the results revealed 6 associated genera in the adenovirus group, 5 associated genera in the cytomegalovirus group, 4 associated genera in the EBV group, 3 associated genera in the H1N1 group and 5 associated genera in the HSV-1 group. The causal relationship between the GM and VIDs provides new ideas and a pretheoretical basis for exploring microbiota transplantation as a strategy for treating VID.

## 1. Introduction

Every year, viral infections cause many illnesses and even deaths, as well as considerable economic losses.^[1]^ There have been pandemics caused by viruses in human history, including the 1918 influenza pandemic (50 million deaths) and the current HIV/AIDS pandemic (36 million deaths to date).^[2]^ To date, viral infections continue to cause extremely high levels of morbidity and mortality globally, which can be attributed to complex and multifactorial external drivers, including changes in environmental, biological, socioeconomic and political factors, as well as the expansion of international travel and trade.^[3–5]^ Therefore, we must actively seek ways and strategies to stop the development of VIDs or to develop effective prevention and treatment strategies to reduce their morbidity and mortality.

The organisms that cause viral infections are viruses, both DNA and RNA viruses, which mutate rapidly and whose evolution is inextricably linked to ecological behavior.^[6]^ Viruses also have a variety of transmission routes and pathogenic mechanisms. Severe acute respiratory syndrome coronavirus 2, the causative agent of COVID-19, has spread so rapidly and widely that it has triggered global healthcare and financial crises.^[7]^ AIDS is transmitted by HIV through blood, mother-to-child, and sexual transmission, and HIV attacks the body’s CD4+ T cells, resulting in a defective immune system.^[8,9]^ Herpes simplex encephalitis is the most common form of sporadic viral encephalitis caused by Herpes simplex virus 1 (HSV-1) infection via the olfactory bulb during primary infection or viral latency reactivation from the trigeminal ganglion.^[10,11]^ Human cytomegalovirus (HCMV, also known as human herpesvirus 5) is a typical member of the *Beta herpesvirinae* family that is latent, replicates profusely when the host’s immune system is compromised and causes severe visceral disease but is otherwise largely asymptomatic.^[12]^ Epstein-Barr virus (EBV) is a ubiquitous oncogenic virus associated with many different human malignancies and autoimmune diseases.^[13,14]^ Human adenovirus (AdV) is a DNA virus that usually causes mild infections of the upper or lower respiratory tract, gastrointestinal tract, or conjunctiva, and young children are commonly infected due to a lack of humoral immunity.^[15]^

The gut microbiota (GM) is a large and complex community of microorganisms in the gut. Currently, GM has been implicated as a risk or preventive factor for a variety of diseases, including infectious diseases.^[16,17]^ However, owing to objective factors such as technology, research methods, and ethics, early diagnosis and prognostic strain screening remain barriers to research. Therefore, most current studies are based on observing the composition and changes in the GM in patients’ feces and verifying the experimental results of transplanting the GM into probiotic mice, which are influenced by various factors, such as the living environment, diet, and antibiotic use.^[18–20]^

In summary, the specific mechanisms by which the GM influences viral infectious disease (VID) are unknown. Therefore, it is crucial to explore the causal relationship between GM and VID. To deepen our understanding of VID, we further explored the causal relationship between the GM and 5 VIDs, including AdV, CMV, EBV, influenza virus subtype H1N1 (H1N1) and herpes simplex virus 1 infection, via MR. Mendelian randomization (MR) is a research method based on pooled data from genome-wide association studies (GWAS) used to explore whether there is a causal relationship between exposure factors and outcome factors.^[21]^ The underlying assumption is that if a genetic variant is associated with an increased likelihood of a specific exposure (e.g., a particular lifestyle factor or a biomarker level), and this genetic variant is also associated with the outcome of interest, then it can be inferred that the exposure has a causal impact on the outcome.^[22,23]^ The data of GWAS is large-scale and comprehensive, providing a large number of potential instrumental variables for MR analyses. This has led to the emergence of MR as a powerful tool. MR has made significant contributions to epidemiological studies of various diseases. Firstly, compared with traditional observational studies, it has a remarkable ability to eliminate confounding factors, which enables more accurate and reliable estimations of the causal effects between exposure factors and outcomes and also makes the research results more stable. Secondly, MR has broadened the scope of research questions that can be explored in epidemiology. In addition, MR helps to identify potential drug targets and evaluate the causal roles of biological pathways in the development of diseases.^[24,25]^

This study performed a 2-sample MR analysis via the the MRC Integrative Epidemiology Unit Open GWAS program to investigate the causal relationships between GM and a variety of VIDs, including AdV, CMV, EBV, H1N1, and HSV-1 infections. By applying MR methods, we can explore whether GM arbitrarily influences the risk of VID. On this basis, we aimed to elucidate the role of GM in the pathogenesis of VID and to inform the development of new therapeutic strategies for VID.

## 2. Materials and methods

### 2.1. Study design

**Figure 1.**
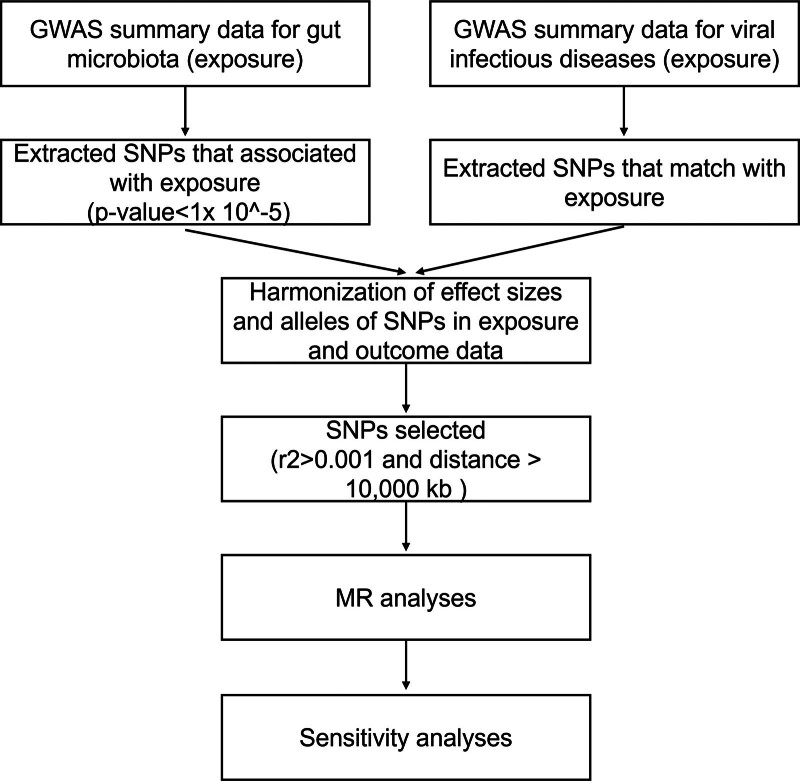
Flowchart illustrating the present MR study. GWAS = genome-wide association studies, MR = Mendelian randomization, SNPs = single nucleotide polymorphisms.

In this work, 2-sample MR was used to assess the association between GM and VID. The flowchart of the study is shown in Figure 1. Three core assumptions of standard MR were used to make the MR results convincing: the selected IVs must be significantly associated with GM taxa, the IVs included in MR analysis did not correlate with the confounders that affected both the GM and VID, and there was no other connection between IVs and VID, except for the influence of the GM.

### 2.2. Data sources of GM and VID

The genetic data for the gut microbiome came from the latest GWAS summary data, in which the MiBioGen consortium curated and analyzed genome-wide genotypes and 16S fecal microbiome data from 18,340 individuals (24 cohorts). The GWAS summary data included a total of 211 GM taxa (131 genera, 35 families, 20 orders, 16 classes, and 9 phyla). We selected data from 131 genera for subsequent analysis. The GWAS summary data of AdV, H1N1, HSV-1, CMV and EBV comprised 3283 cases and 1,81,704 controls. The present study is a secondary analysis of publicly available GWAS summary statistics. Ethical approval was granted for each of the original GWAS. In addition, no individual-level data was used in this study. Therefore, no new ethical review board approval was needed.

### 2.3. Instrumental variable selection

First, we selected the single nucleotide polymorphisms (SNPs) with significant associations with the GM (*P* < 1 × 10^−5^). We subsequently excluded the SNPs with linkage disequilibrium in the analysis. The linkage disequilibrium of the chosen SNPs strongly related to the GM should meet the conditions that *r*^2^ < 0.001 and a distance >10,000 kb. An important step in MR analysis is to ensure that the effects of SNPs on exposure correspond to the same allele as the effects on outcome. After matching the outcome, we removed palindromic SNPs.

We extracted the relevant information: chromosome, effect allele, other allele, effect allele frequency, effect size (β), standard error, and *P* value. Finally, we calculated the explained variance (*R*^2^) and *F*-statistic parameters to determine whether the identified IVs were strongly associated with exposure. Generally, SNPs with *F*-statistic parameters <10 are considered weak instruments.

### 2.4. Statistical analysis

The MR approach employs 5 analytical methods, including MR-Egger, the weighted median, inverse variance weighting, a simple model, and a weighted model, to investigate the causal relationship between GM and VID. To improve the accuracy of the MR analysis results, we performed sensitivity analyses, including heterogeneity and pleiotropy analyses, using Cochrane’s *Q* test calculated via the inverse variance weighted methods and the MR-Egger intercept test, respectively. All analyses were performed in the R 4.3.2 environment with the R package “TwoSampleMR.” A significance threshold of *P* < .05 was applied.

## 3. Results

### 3.1. Instrumental variable selection

According to the selection criteria for IVs, 1531 SNPs were used as IVs for 131 bacterial genera. Details about the selected instrumental variables are shown in an additional file: Table S1, Supplemental Digital Content, https://links.lww.com/MD/P353. An overview of the sources of the virus infection disease data is shown in Table 1.

**Table 1:** Overview of the source of virus infection diseases data.

GWAS ID	Trait	Data source	Cases	Population
prot-a-261	Adenovirus	IEU Open GWAS	3301	European
ieu-b-4900	Cytomegalovirus	IEU Open GWAS	5010	European
ebi-a-GCST006346	Herpes simplex virus 1	IEU Open GWAS	645	European
ieu-b-4901	Epstein-Barr virus	IEU Open GWAS	5010	European
ieu-b-4903	Influenza virus subtype H1N1	IEU Open GWAS	683	European

GWAS = genome-wide association studies, IEU = the MRC Integrative Epidemiology Unit.

### 3.2. Causal effects of the GM on multiple VID types

#### 3.2.1. AdV

**Figure 2.**
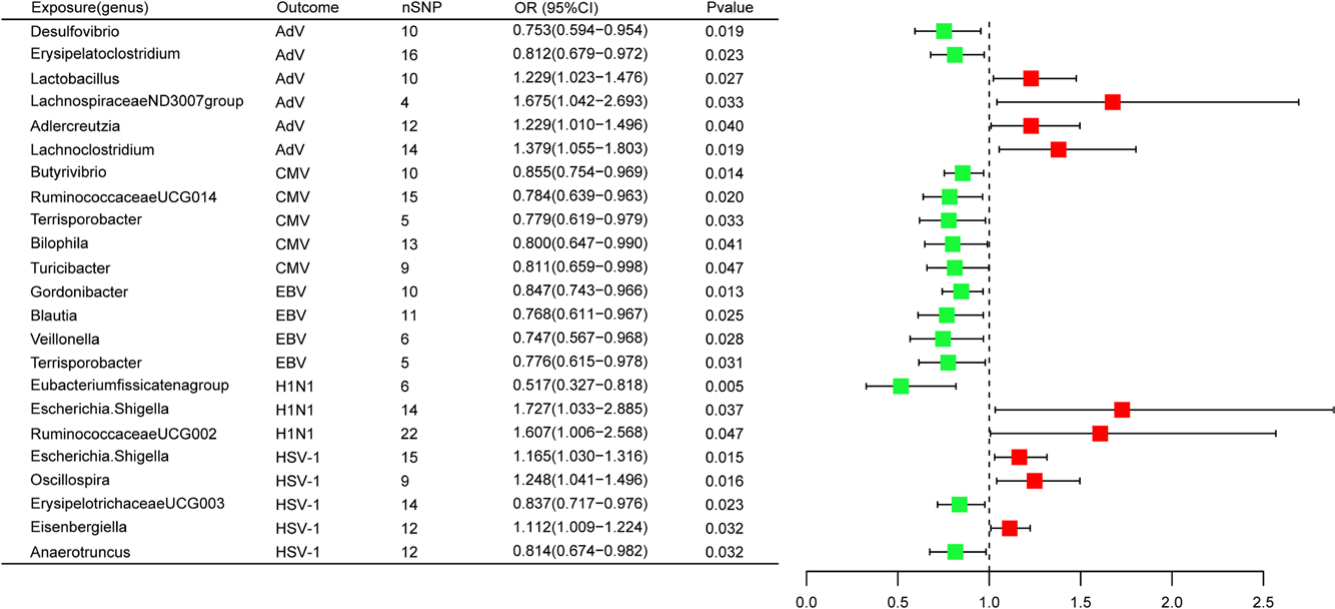
Forest plot of Mendelian randomization estimates the relationship between the gut microbiota and viral infectious diseases. This plot shows IVW estimates for gut microbiota taxa significantly associated with viral infectious diseases. Blocks represent IVW estimates, and black bars represent 95% confidence intervals for IVW estimates. OR > 1 (red blocks) indicates increased risk, and OR < 1 (green blocks) indicates decreased risk. AdV = adenovirus, CI = confidence interval, CMV = cytomegalovirus, EBV = Epstein-Barr virus, H1N1 = influenza virus subtype H1N1, HSV-1 = herpes simplex virus 1, IVW = inverse variance weighted, OR = odds ratio, nSNP = number of single nucleotide polymorphisms.

A total of 6 GM genera were associated with AdV. As shown in Figure 2 and the additional file: Table S2, Supplemental Digital Content, https://links.lww.com/MD/P354, MR analysis suggested that genetic prediction of 4 GM s (genus *Lactobacillus*, genus LachnospiraceaeND3007group, genus *Adlercreutzia* and genus *Lachnoclostridium*) was associated with an increased risk of AdV. The genus *Lactobacillus* (odds ratio [OR] = 1.229, 95% CI = 1.023–1.476, *P* = .027), genus LachnospiraceaeND3007group (OR = 1.675, 95% CI = 1.042–2.693, *P* = .033), genus *Adlercreutzia* (OR = 1.229, 95% CI = 1.010–1.496, *P* = .040), genus *Lachnoclostridium* (OR = 1.379, 95% CI = 1.055–1.803, *P* = .019) significantly increased the risk of AdV. Genetic prediction of 2 GM s (genus *Desulfovibrio* and genus *Erysipelatoclostridium*) was associated with a decreased risk of AdV. The genus *Desulfovibrio* (OR = 0.753, 95% CI = 0.594–0.954, *P* = .019), and genus Erysipelatoclostridium (OR = 0.812, 95% CI = 0.679–0.972, *P* = .023) significantly decreased the risk of AdV.

#### 3.2.2. CMV

A total of 5 GM were associated with CMV. As shown in Figure 2 and additional file: Table S2, Supplemental Digital Content, https://links.lww.com/MD/P354, MR analysis suggested that genetic prediction of all 5 GM (genus *Butyrivibrio*, genus RuminococcaceaeUCG014, genus *Terrisporobacter*, genus *Bilophila*, and genus *Turicibacter*) was associated with a decreased risk of CMV. The genera Butyrivibrio (OR = 0.855, 95% CI = 0.754–0.969, *P* = .014), RuminococcaceaeUCG014 (OR = 0.784, 95% CI = 0.639–0.963, *P* = .020), Terrisporobacter (OR = 0.779, 95% CI = 0.619–0.979, *P* = .033), Bilophila (OR = 0.800, 95% CI = 0.647–0.990, *P* = .041), and Turicibacter (OR = 0.811, 95% CI = 0.659–0.998, *P* = .047) significantly decreased the risk of CMV.

#### 3.2.3. EBV

A total of 4 GM were associated with EBV. As shown in Figure 2 and additional file: Table S2, Supplemental Digital Content, https://links.lww.com/MD/P354, MR analysis suggested that genetic prediction of all 4 GM (genus *Gordonibacter*, genus *Blautia*, genus *Veillonella*, and genus *Terrisporobacter*) was associated with a decreased risk of EBV. The genera Gordonibacter (OR = 0.847, 95% CI = 0.743–0.966, *P* = .013), Blautia (OR = 0.768, 95% CI = 0.611–0.967, *P* = .025), Veillonella (OR = 0.747, 95% CI = 0.567–0.968, *P* = .028) and Terrisporobacter (OR = 0.776, 95% CI = 0.615–0.978, *P* = .028) significantly decreased the risk of EBV.

#### 3.2.4. H1N1

A total of 3 GM were associated with H1N1. As shown in Figure 2 and the additional file: Table S2, Supplemental Digital Content, https://links.lww.com/MD/P354, MR analysis suggested that genetic prediction of 2 GM (genus *Escherichia Shigella* and the genus RuminococcaceaeUCG002) were associated with an increased risk of H1N1. The genus *E Shigella* (OR = 1.727, 95% CI = 1.033–2.885, *P* = .037) and the genus RuminococcaceaeUCG002 (OR = 1.607, 95% CI = 1.006–2.568, *P* = .047) significantly increased the risk of H1N1. Genetic prediction of 1 GM, the genus Eubacterium fissicatena group (OR = 0.517, 95% CI = 0.327–0.818, *P* = .005) was associated with a decreased risk of H1N1.

#### 3.2.5. HSV-1

A total of 5 GM were associated with HSV-1. As shown in Figure 2 and the additional file: Table S2, Supplemental Digital Content, https://links.lww.com/MD/P354, MR analysis suggested that the genetic prediction of 3 GM (genus *E Shigella*, the genus *Oscillospira*, and the genus *Eisenbergiella*) was associated with an increased risk of HSV-1. The genus *E Shigella* (OR = 1.165, 95% CI = 1.030–1.316, *P* = .015), the genus *Oscillospira* (OR = 1.248, 95% CI = 1.041–1.496, *P* = .016) and the genus *Eisenbergiella* (OR = 1.112, 95% CI = 1.009–1.224, *P* = .032) significantly increased the risk of HSV-1. Genetic prediction of 2 GM (genus ErysipelotrichaceaeUCG003 and genus *Anaerotruncus*) was associated with a decreased risk of HSV-1. The genera ErysipelotrichaceaeUCG003 (OR = 0.837, 95% CI = 0.717–0.976, *P* = .023) and Anaerotruncus (OR = 0.814, 95% CI = 0.674–0.982, *P* = .032) significantly decreased the risk of HSV-1.

### 3.3. Sensitivity analyses

According to the MR-Egger regression intercept approach, genetic pleiotropy did not bias the results, and there was no horizontal pleiotropy in the MR study (*P* > .05, Additional file: Table S3, Supplemental Digital Content, https://links.lww.com/MD/P355). Cochran’s *Q* tests revealed no significant heterogeneity (*P* > .05, Additional file: Table S3, Supplemental Digital Content, https://links.lww.com/MD/P355). The results of the “leave-one-out” analysis proved that MR analysis was reliable. (The null line is not within the total confidence interval of the SNPs. Additional file: Figures S1–S15, Supplemental Digital Content, https://links.lww.com/MD/P356) The scatter plots show the overall effect of the GM on VID (Additional file: Figures S1–S15, Supplemental Digital Content, https://links.lww.com/MD/P356). In addition, the forest plots revealed causal associations between the GM and VID (Additional file: Figures S1–S15, Supplemental Digital Content, https://links.lww.com/MD/P356).

## 4. Discussion

Our MR study revealed a possible causal relationship between the genetic risk of 22 GMs and 5 types of VID. In particular, we found that certain GM (*E Shigella*) significantly increased the risk of HSV-1 and H1N1 infection.

Adenovirus is an enveloped, icosahedral, double-stranded DNA virus that can cause mild to severe diseases such as respiratory infections, epidemic conjunctivitis, and gastroenteritis.^[26]^ Research has shown that probiotics, such as *Lactobacillus*, LactobacillusND3007, and *Lactobacillus acidophilus*, can alleviate diseases by regulating immune activity.^[27,28]^ Similarly, studies have shown a positive correlation between the overgrowth of Desulfovibrio and various human diseases.^[29]^ Milosavljevic MN et al^[30]^ confirmed the clinical relevance of Erysipelatoclostridium as a cause of several severe infections, mainly in immunocompromised inpatients. Cytomegaloviruses can cause a very large variety of clinical syndromes in any part of the gastrointestinal tract, the central or peripheral nervous system, and the eyes.^[31]^ Shimizu et al^[32]^ speculated that the invasion of cytomegalovirus into the mucosal layer induces a strong immune response and plays a role in the development of chronic inflammatory damage, but no correlation with the GM has been identified. EBV is a human tumor virus that is etiologically linked to various malignancies.^[33]^ Supplementing with *Lactobacillus casei* can reduce the plasma levels of EBV and CMV antibodies, which may enhance host immunity.^[34]^ Wang et al^[35]^ One study reported that *Firmicutes* and *Lactobacillus* were increased in the EBV-infected group, but there was no mention of any association between the GM and reduced EBV risk. H1N1 is the most common type of influenza virus in humans. Despite its complete genome sequence, the evolution and mutations of H1N1 are still unclear.^[36]^ The relationship between gut microbes and H1N1 infections is not fully understood.^[37]^ HSV-1 is a prevalent human virus that is a latent infection in the majority of the global population.^[38]^ As of 2020, an estimated half a billion people had genital infection with HSV type 2 or type 1, and several billion had oral HSV type 1 infection.^[39]^ Research has shown that changes in GM and HSV infection are factors leading to disease development.^[40]^

Our MR study evaluated the causal relationship between GM and VIDs. We identified 254 SNPs via the GWAS dataset and different models to determine their causal relationships. In our analysis, we identified directional pleiotropy and adjusted it by applying the MR-PRESSO test after excluding all suspicious outliers. Therefore, the results from the IV random effects models were selected. In our study, we observed a strong causal relationship between GM and VIDs. Sensitivity testing supports the stability and accuracy of causal results. Our research results provide evidence for the corresponding associations between several GM and several VIDs. For VIDs, early prevention and clinical intervention can be achieved by changing the type and quantity of the GM.

Although this study has implications for clinical practice, some limitations should be noted. First, the MR analysis method used in this study relies on genetic variation, which can reflect only the effect of genetic variation at the population level and does not consider the effects of individual differences. Second, we selected only 5 VIDs, and our conclusions cannot be generalized to all infectious diseases.

## 5. Conclusion

In summary, there is a causal relationship between GM and VIDs. Our findings suggest that individuals with a history of infectious diseases require special clinical attention to changes in the GM to prevent the development of potential VIDs. Further research is needed to examine the biological mechanisms behind this association.

## Acknowledgments

We acknowledge the researchers and participants of the GWAS used in our study.

## Author contributions

**Conceptualization:** Xudong Liu.

**Methodology:** Sixia Jiang.

**Project administration:** Sixia Jiang.

**Resources:** Hao Hu.

**Software:** Hao Hu, Ya Song.

**Supervision:** Ya Song, Sijie Zhu.

**Validation:** Junjun He, Shirui Yu.

**Visualization:** Junjun He.

**Writing – original draft:** Hao Hu, Feng Xie, Shirui Yu.

**Writing – review & editing:** Hao Hu, Xudong Liu.

## Supplementary Material



## References

[1] Yeh YT Nisic M Yu X Xia Y Zheng SY. Point-of-care microdevices for blood plasma analysis in viral infectious diseases. Ann Biomed Eng. 2014;42:2333–43. 24879614 10.1007/s10439-014-1044-2 PMC7088150

[2] Parks T Hill AV Chapman SJ. The perpetual challenge of infectious diseases. N Engl J Med. 2012;367:90. 10.1056/NEJMc1204960 22762339

[3] Morens DM Fauci AS. Emerging infectious diseases: threats to human health and global stability. PLoS Pathog. 2013;9:e1003467. 23853589 10.1371/journal.ppat.1003467 PMC3701702

[4] Khan K Arino J Hu W. Spread of a novel influenza A (H1N1) virus via global airline transportation. N Engl J Med. 2009;361:212–4. 19564630 10.1056/NEJMc0904559

[5] Wilson ME. The traveller and emerging infections: sentinel, courier, transmitter. J Appl Microbiol. 2003;94(Suppl):1S–11S. 12675931 10.1046/j.1365-2672.94.s1.1.x

[6] Pybus OG Rambaut A. Evolutionary analysis of the dynamics of viral infectious disease. Nat Rev Genet. 2009;10:540–50. 19564871 10.1038/nrg2583 PMC7097015

[7] Ochani R Asad A Yasmin F. COVID-19 pandemic: from origins to outcomes. A comprehensive review of viral pathogenesis, clinical manifestations, diagnostic evaluation, and management. Infez Med. 2021;29:20–36. 33664170

[8] Kelschenbach J He H Kim BH. Efficient expression of HIV in immunocompetent mouse brain reveals a novel nonneurotoxic viral function in hippocampal synaptodendritic injury and memory impairment. mBio. 2019;10:e00591–19. 31266862 10.1128/mBio.00591-19 PMC6606797

[9] Xing J Wu F Wang S Krensky AM Mody CH Zheng C. Granulysin production and anticryptococcal activity is dependent upon a far upstream enhancer that binds STAT5 in human peripheral blood CD4+ T cells. J Immunol. 2010;185:5074–81. 20889547 10.4049/jimmunol.1001725 PMC6959525

[10] Whitley RJ. Herpes simplex encephalitis: adolescents and adults. Antiviral Res. 2006;71:141–8. 16675036 10.1016/j.antiviral.2006.04.002

[11] Zhu H Zheng C. The race between host antiviral innate immunity and the immune evasion strategies of herpes simplex virus 1. Microbiol Mol Biol Rev. 2020;84:e00099–20. 32998978 10.1128/MMBR.00099-20 PMC7528619

[12] Griffiths P Reeves M. Pathogenesis of human cytomegalovirus in the immunocompromised host. Nat Rev Microbiol. 2021;19:759–73. 34168328 10.1038/s41579-021-00582-z PMC8223196

[13] Damania B Kenney SC Raab-Traub N. Epstein-Barr virus: biology and clinical disease. Cell. 2022;185:3652–70. 36113467 10.1016/j.cell.2022.08.026 PMC9529843

[14] Moin AT Patil RB Tabassum T. Immunoinformatics approach to design novel subunit vaccine against the Epstein-Barr virus. Microbiol Spectr. 2022;10:e0115122. 36094198 10.1128/spectrum.01151-22 PMC9603631

[15] Lynch JP 3rd Fishbein M Echavarria M. Adenovirus. Semin Respir Crit Care Med. 2011;32:494–511. 21858752 10.1055/s-0031-1283287

[16] Long Y Tang L Zhou Y Zhao S Zhu H. Causal relationship between gut microbiota and cancers: a two-sample Mendelian randomisation study. BMC Med. 2023;21:66. 36810112 10.1186/s12916-023-02761-6 PMC9945666

[17] Wiertsema SP van Bergenhenegouwen J Garssen J Knippels LMJ. The interplay between the gut microbiome and the immune system in the context of infectious diseases throughout life and the role of nutrition in optimizing treatment strategies. Nutrients. 2021;13:886. 33803407 10.3390/nu13030886 PMC8001875

[18] Cai Y Chen L Zhang S Zeng L Zeng G. The role of gut microbiota in infectious diseases. WIREs Mech Dis. 2022;14:e1551. 34974642 10.1002/wsbm.1551

[19] Yeoh YK Zuo T Lui GC. Gut microbiota composition reflects disease severity and dysfunctional immune responses in patients with COVID-19. Gut. 2021;70:698–706. 33431578 10.1136/gutjnl-2020-323020 PMC7804842

[20] Zhou A Yuan Y Yang M. Crosstalk between the gut microbiota and epithelial cells under physiological and infectious conditions. Front Cell Infect Microbiol. 2022;12:832672. 35155283 10.3389/fcimb.2022.832672 PMC8829037

[21] Sekula P Del Greco MF Pattaro C Köttgen A. Mendelian randomization as an approach to assess causality using observational data. J Am Soc Nephrol. 2016;27:3253–65. 27486138 10.1681/ASN.2016010098 PMC5084898

[22] Zheng Q Lin R Wang D Zheng C Xu W. Effects of circulating inflammatory proteins on spinal degenerative diseases: evidence from genetic correlations and Mendelian randomization study. JOR Spine. 2024;7:e1346. 38895179 10.1002/jsp2.1346 PMC11183170

[23] Zheng Q Wang D Lin R. Mendelian randomization analysis suggests no associations of human herpes viruses with amyotrophic lateral sclerosis. Front Neurosci. 2023;17:1299122. 38156274 10.3389/fnins.2023.1299122 PMC10754516

[24] Xiang K Wang P Xu Z. Causal effects of gut microbiome on systemic lupus erythematosus: a two-sample Mendelian randomization study. Front Immunol. 2021;12:667097. 34557183 10.3389/fimmu.2021.667097 PMC8453215

[25] Smith GD Ebrahim S. “Mendelian randomization”: can genetic epidemiology contribute to understanding environmental determinants of disease? Int J Epidemiol. 2003;32:1–22. 12689998 10.1093/ije/dyg070

[26] Wasimuddin Corman VM Ganzhorn JU Rakotondranary J Ratovonamana YR Drosten C. Adenovirus infection is associated with altered gut microbial communities in a non-human primate. Sci Rep. 2019;9:13410. 31527752 10.1038/s41598-019-49829-z PMC6746978

[27] Debnath N Kumar A Yadav AK. Probiotics as a biotherapeutics for the management and prevention of respiratory tract diseases. Microbiol Immunol. 2022;66:277–91. 35462444 10.1111/1348-0421.12980

[28] Liu X Jiang W Lu G. The potential role of pyrroloquinoline quinone to regulate thyroid function and gut microbiota composition of graves’ disease in mice. Pol J Microbiol. 2023;72:443–60. 38095308 10.33073/pjm-2023-042 PMC10725160

[29] Singh SB Carroll-Portillo A Lin HC. Desulfovibrio in the gut: the enemy within? Microorganisms. 2023;11:1772. 37512944 10.3390/microorganisms11071772 PMC10383351

[30] Milosavljevic MN Kostic M Milovanovic J. Antimicrobial treatment of Erysipelatoclostridium ramosum invasive infections: a systematic review. Rev Inst Med Trop Sao Paulo. 2021;63:e30. 33852713 10.1590/S1678-9946202163030 PMC8046505

[31] Schattner A. The wide spectrum of presentations of cytomegalovirus infection in immunocompetent hosts: an exhaustive narrative review. Pathogens. 2024;13:667. 39204267 10.3390/pathogens13080667 PMC11357360

[32] Shimizu M Ohta K Wada H Sumita R Yachie A Koizumi S. Cytomegalovirus-associated protracted diarrhoea in an immunocompetent boy. J Paediatr Child Health. 2006;42:259–62. 16712555 10.1111/j.1440-1754.2006.00851.x

[33] Kanda T Yajima M Ikuta K. Epstein-Barr virus strain variation and cancer. Cancer Sci. 2019;110:1132–9. 30697862 10.1111/cas.13954 PMC6447851

[34] Eladwy RA Vu HT Shah R Li CG Chang D Bhuyan DJ. The fight against the carcinogenic Epstein-Barr virus: gut microbiota, natural medicines, and beyond. Int J Mol Sci . 2023;24:1716. 36675232 10.3390/ijms24021716 PMC9862477

[35] Wang X Sun L Li P Zhang S. Changes in the gut microbiome can predict and decrease Epstein-Barr virus infection risk in children after liver transplantation. Transpl Infect Dis. 2023;25:e14114. 37639316 10.1111/tid.14114

[36] Jiang D Wang Q Bai Z. Could environment affect the mutation of H1N1 influenza virus? Int J Environ Res Public Health. 2020;17:3092. 32365515 10.3390/ijerph17093092 PMC7246512

[37] Lv L Gu S Jiang H. Gut mycobiota alterations in patients with COVID-19 and H1N1 infections and their associations with clinical features. Commun Biol. 2021;4:480. 33850296 10.1038/s42003-021-02036-x PMC8044104

[38] Ramakrishna C Mendonca S Ruegger PM Kim JH Borneman J Cantin EM. Herpes simplex virus infection, acyclovir and IVIG treatment all independently cause gut dysbiosis. PLoS One. 2020;15:e0237189. 32760124 10.1371/journal.pone.0237189 PMC7410316

[39] James C Harfouche M Welton NJ. Herpes simplex virus: global infection prevalence and incidence estimates, 2016. Bull World Health Organ. 2020;98:315–29. 32514197 10.2471/BLT.19.237149 PMC7265941

[40] Protto V Marcocci ME Miteva MT. Role of HSV-1 in Alzheimer’s disease pathogenesis: a challenge for novel preventive/therapeutic strategies. Curr Opin Pharmacol. 2022;63:102200. 35276497 10.1016/j.coph.2022.102200

