# Supplementary material for: Causal relationship between gut microbiota and viral infectious disease: A 2-sample Mendelian randomization study
Source: Medicine (Baltimore). 2025 Jul 4;104(27):e43258. doi: 10.1097/MD.0000000000043258 (PMC12237378; doi:10.1097/MD.0000000000043258)
Supplement: Supplementary file 4 [file medi-104-e43258-s004.docx]

**Additional Figures**

**Figure S1.** MR leave-one-out sensitivity analysis for Gut microbiota on AdV.

**Figure S2.** MR leave-one-out sensitivity analysis for Gut microbiota on CMV.

**Figure S3.** MR leave-one-out sensitivity analysis for Gut microbiota on EBV.

**Figure S4.** MR leave-one-out sensitivity analysis for Gut microbiota on H1N1.

**Figure S5.** MR leave-one-out sensitivity analysis for Gut microbiota on HSV-1.

**Figure S6.** Scatter plots for the effect of Gut microbiota on AdV.

**Figure S7.** Scatter plots for the effect of Gut microbiota on CMV.

**Figure S8.** Scatter plots for the effect of Gut microbiota on EBV.

**Figure S9.** Scatter plots for the effect of Gut microbiota on H1N1.

**Figure S10.** Scatter plots for the effect of Gut microbiota on HSV-1.

**Figure S11.** Forest plots for the effect of Gut microbiota on AdV.

**Figure S12.** Forest plots for the effect of Gut microbiota on CMV.

**Figure S13.** Forest plots for the effect of Gut microbiota on EBV.

**Figure S14.** Forest plots for the effect of Gut microbiota on H1N1.

**Figure S15.** Forest plots for the effect of Gut microbiota on HSV-1.

**Figure S1.** MR leave-one-out sensitivity analysis for Gut microbiota on AdV.


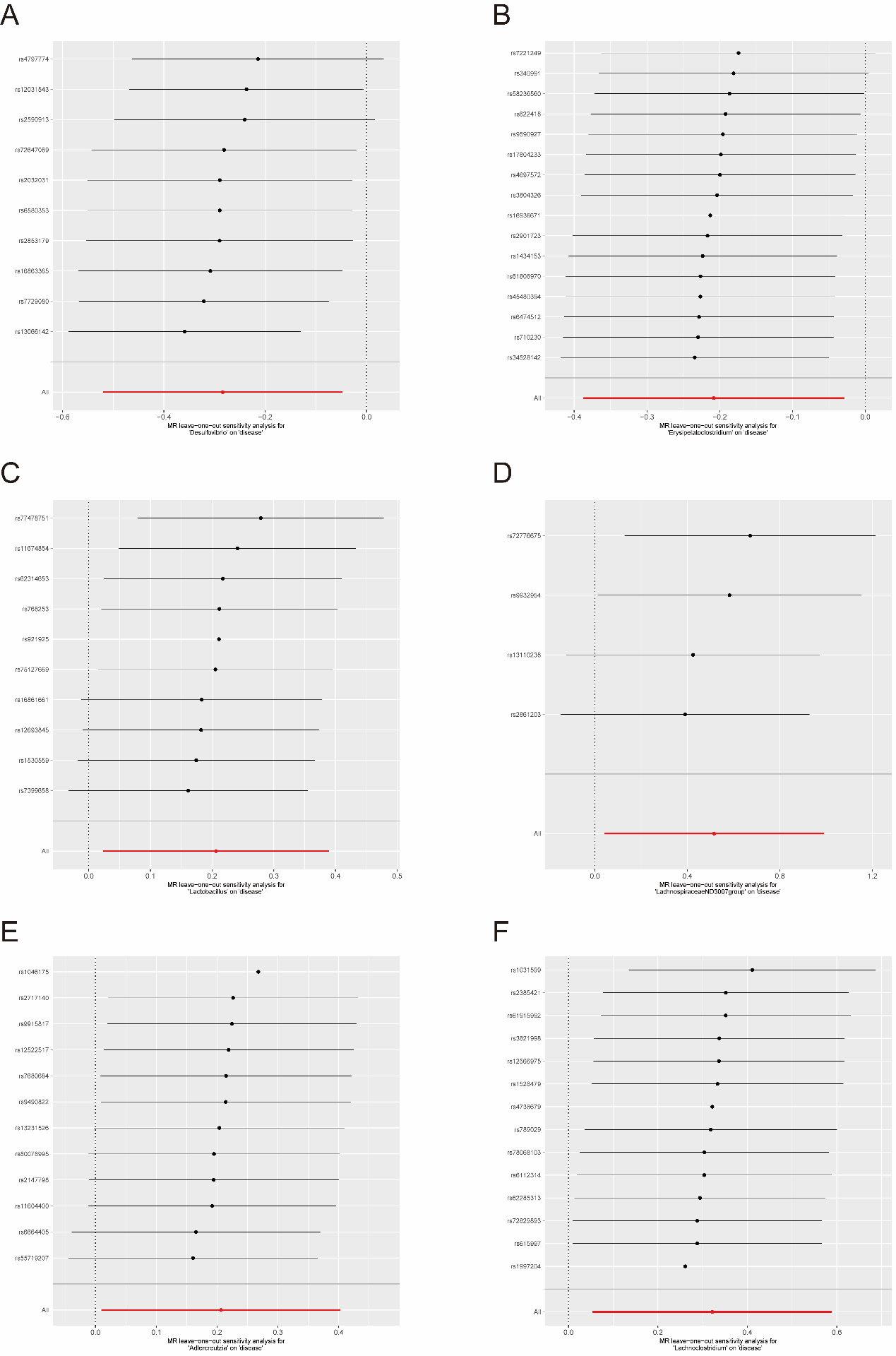


1. Analysis for "genus Desulfovibrio" on "AdV"

(B) Analysis for "genus Erysipelatoclostridium" on "AdV"

(C) Analysis for "genus Lactobacillus" on "AdV"

(D) Analysis for "genus LachnospiraceaeND3007group" on "AdV"

(E) Analysis for "genus Adlercreutzia" on "AdV"

(F) Analysis for "genus Lachnoclostridium" on "AdV"

**Figure S2.** MR leave-one-out sensitivity analysis for Gut microbiota on CMV.


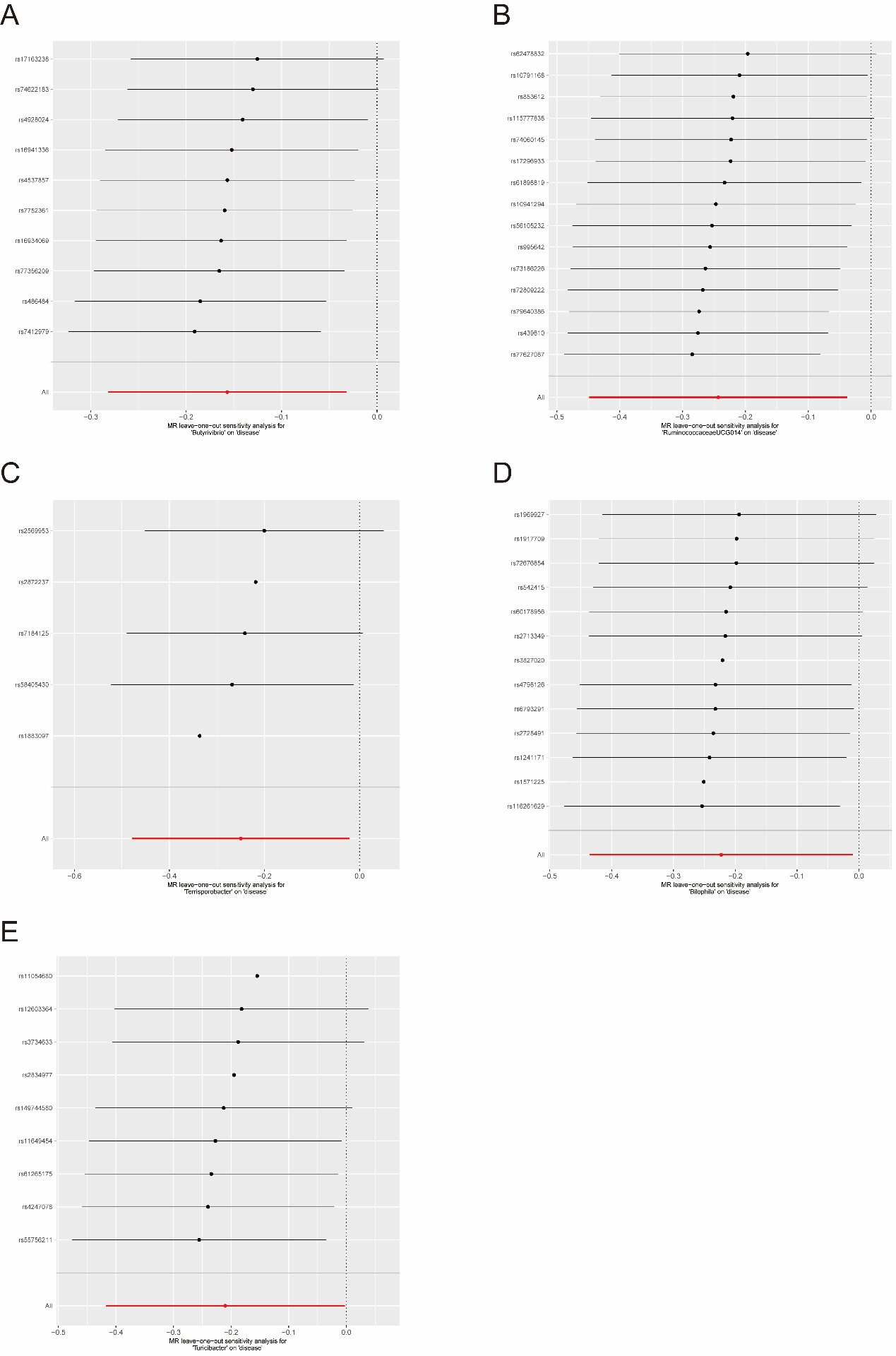


(A) Analysis for "genus Butyrivibrio" on "CMV"

(B) Analysis for "genus RuminococcaceaeUCG014" on "CMV"

(C) Analysis for "genus Terrisporobacter" on "CMV"

(D) Analysis for "genus Bilophila" on "CMV"

(E) Analysis for "genus Turicibacter" on "CMV"

**Figure S3.** MR leave-one-out sensitivity analysis for Gut microbiota on EBV


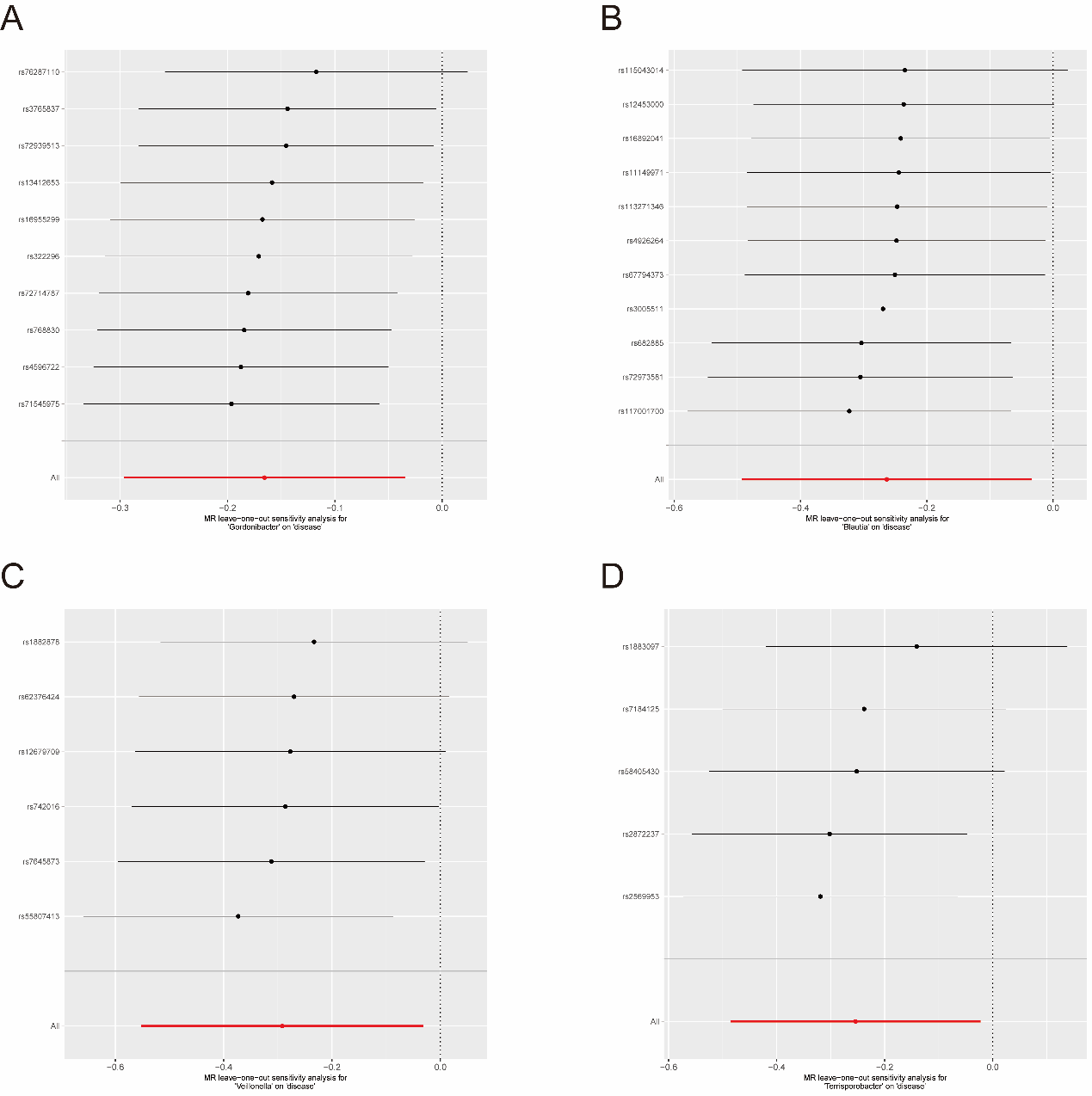


(A) Analysis for "genus Gordonibacter" on "EBV"

(B) Analysis for "genus Blautia" on "EBV"

(C) Analysis for "genus Veillonella" on "EBV"

(D) Analysis for "genus Terrisporobacter" on "EBV"

**Figure S4.** MR leave-one-out sensitivity analysis for Gut microbiota on H1N1.


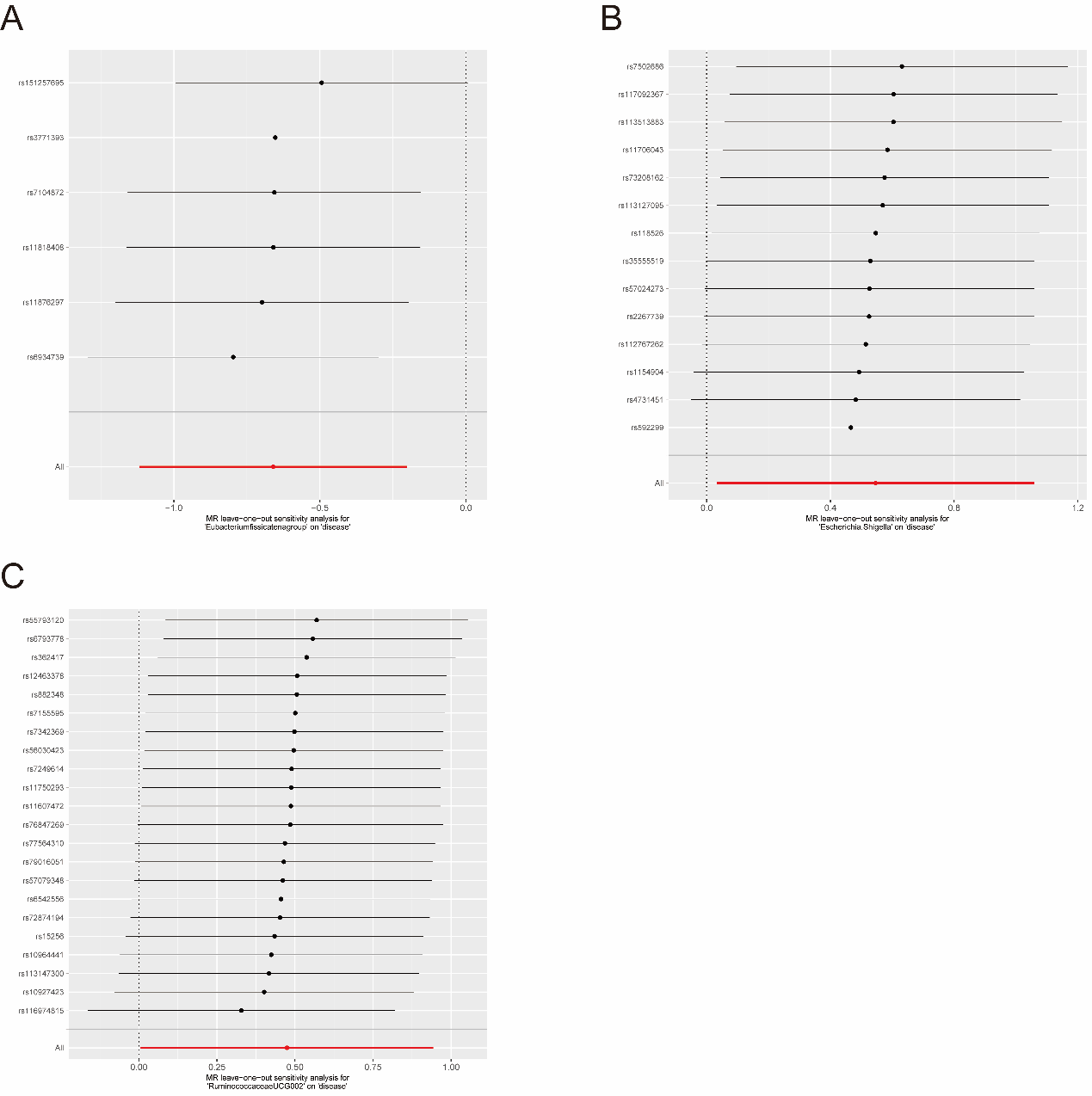


(A) Analysis for "genus Eubacteriumfissicatenagroup" on "H1N1"

(B) Analysis for "genus Escherichia.Shigella" H1N1"

(C) Analysis for "genus RuminococcaceaeUCG002" on "H1N1"

**Figure S5.** MR leave-one-out sensitivity analysis for Gut microbiota on HSV-1.


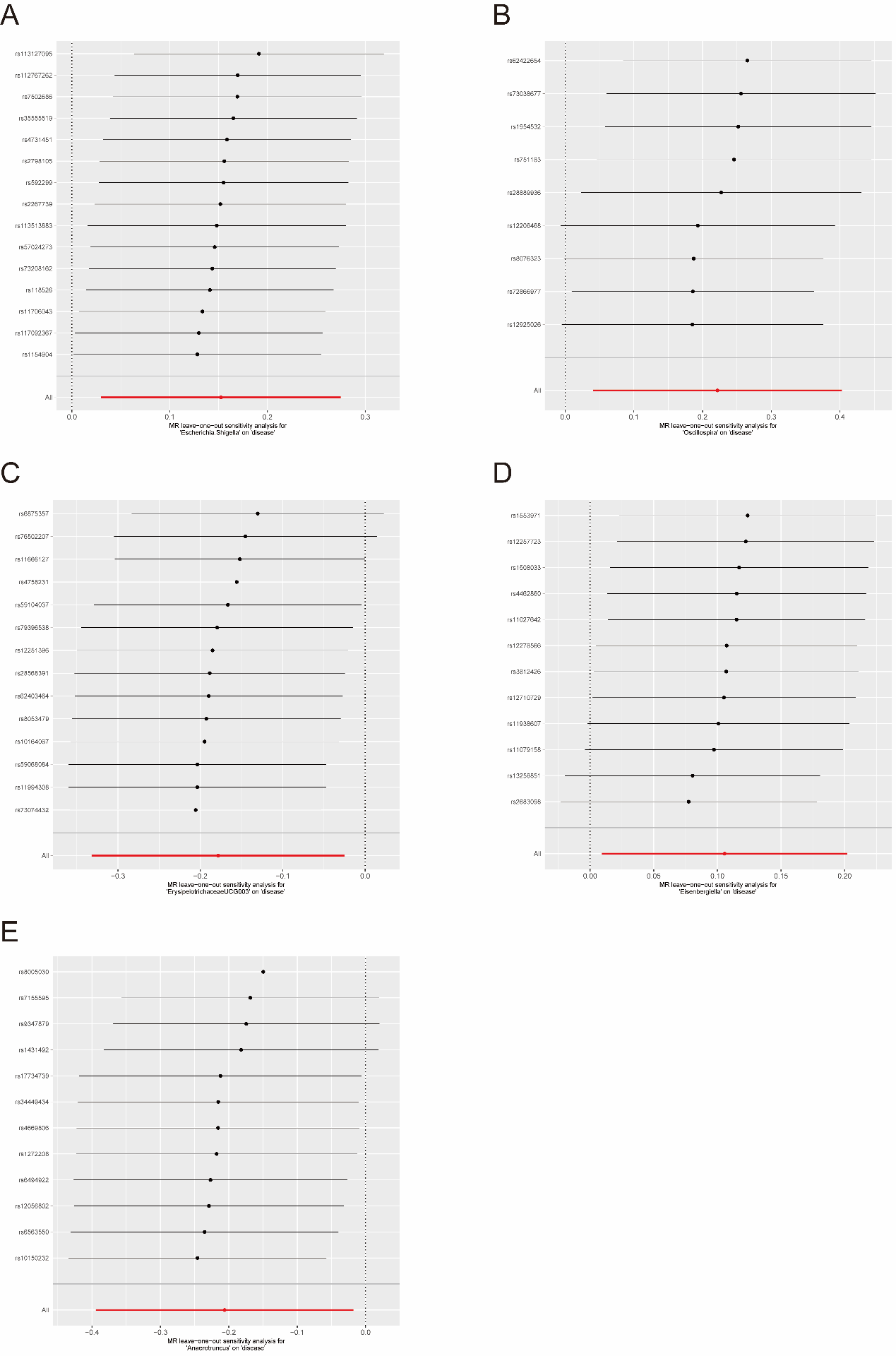


(A) Analysis for "genus Escherichia.Shigella" on " HSV-1"

(B) Analysis for "genus Oscillospira" on "HSV-1"

(C) Analysis for "genus ErysipelotrichaceaeUCG003" on " HSV-1"

(D) Analysis for "genus Eisenbergiella" on " HSV-1"

(E) Analysis for "genus Anaerotruncus" on " HSV-1"

**Figure S6.** Scatter plots for the effect of Gut microbiota on AdV.


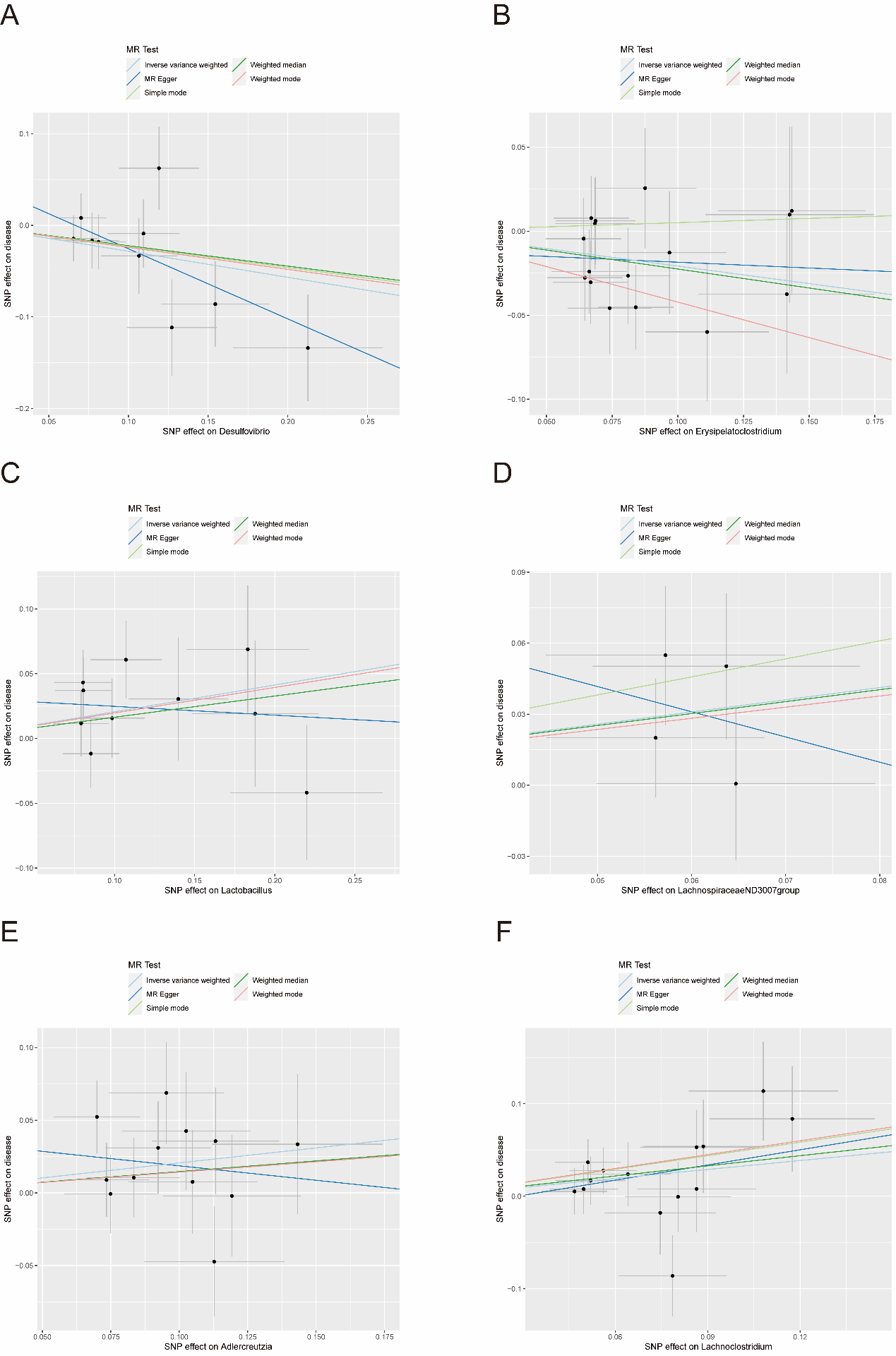


(A) genus Desulfovibrio

(B) genus Erysipelatoclostridium

(C) genus Lactobacillus

(D) genus LachnospiraceaeND3007group

(E) genus Adlercreutzia

(F) genus Lachnoclostridium

**Figure S7.** Scatter plots for the effect of Gut microbiota on CMV.


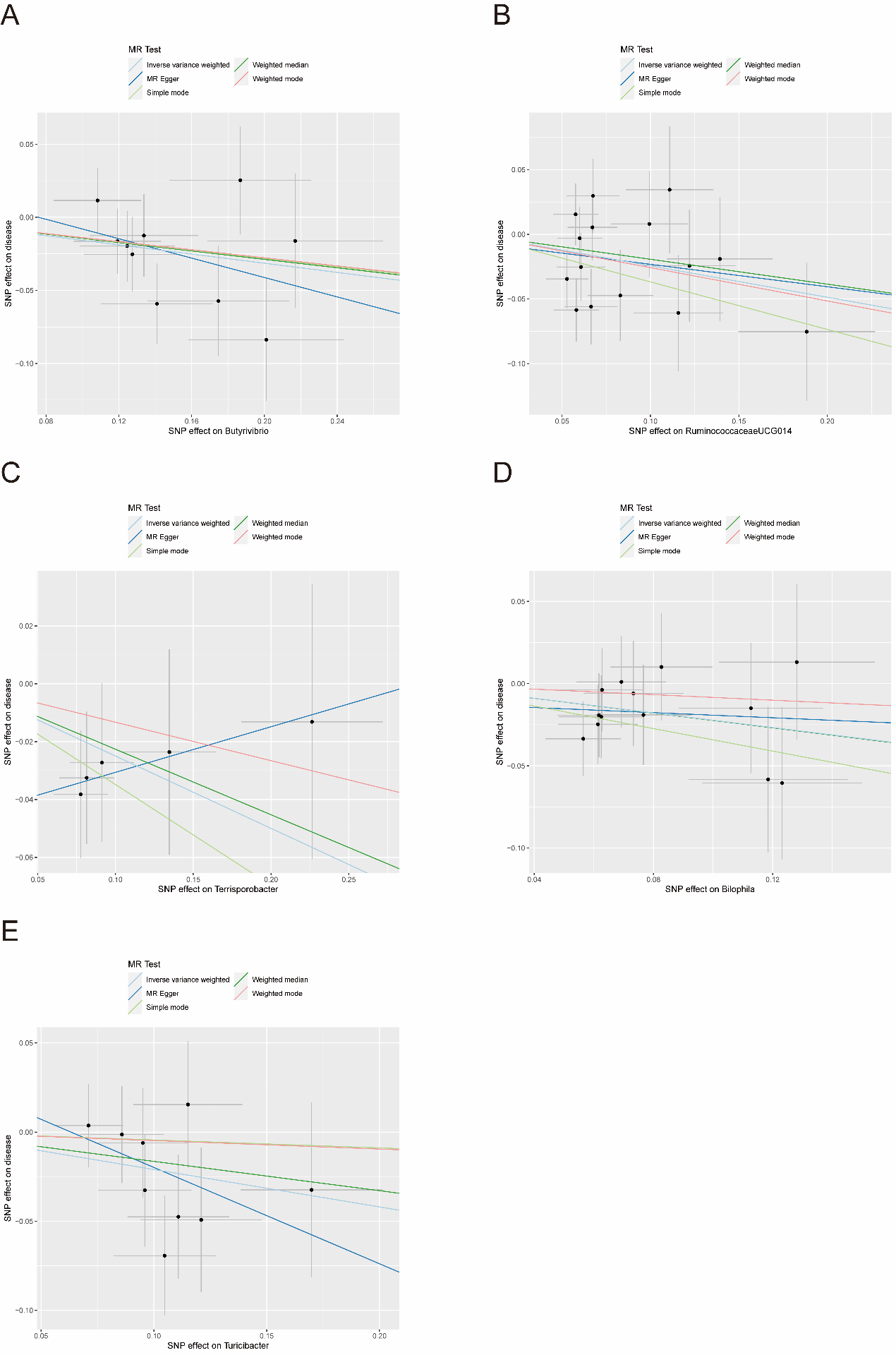


(A) genus Butyrivibrio

(B) genus RuminococcaceaeUCG014

(C) genus Terrisporobacter

(D) genus Bilophila

(E) genus Turicibacter

**Figure S8.** Scatter plots for the effect of Gut microbiota on EBV.


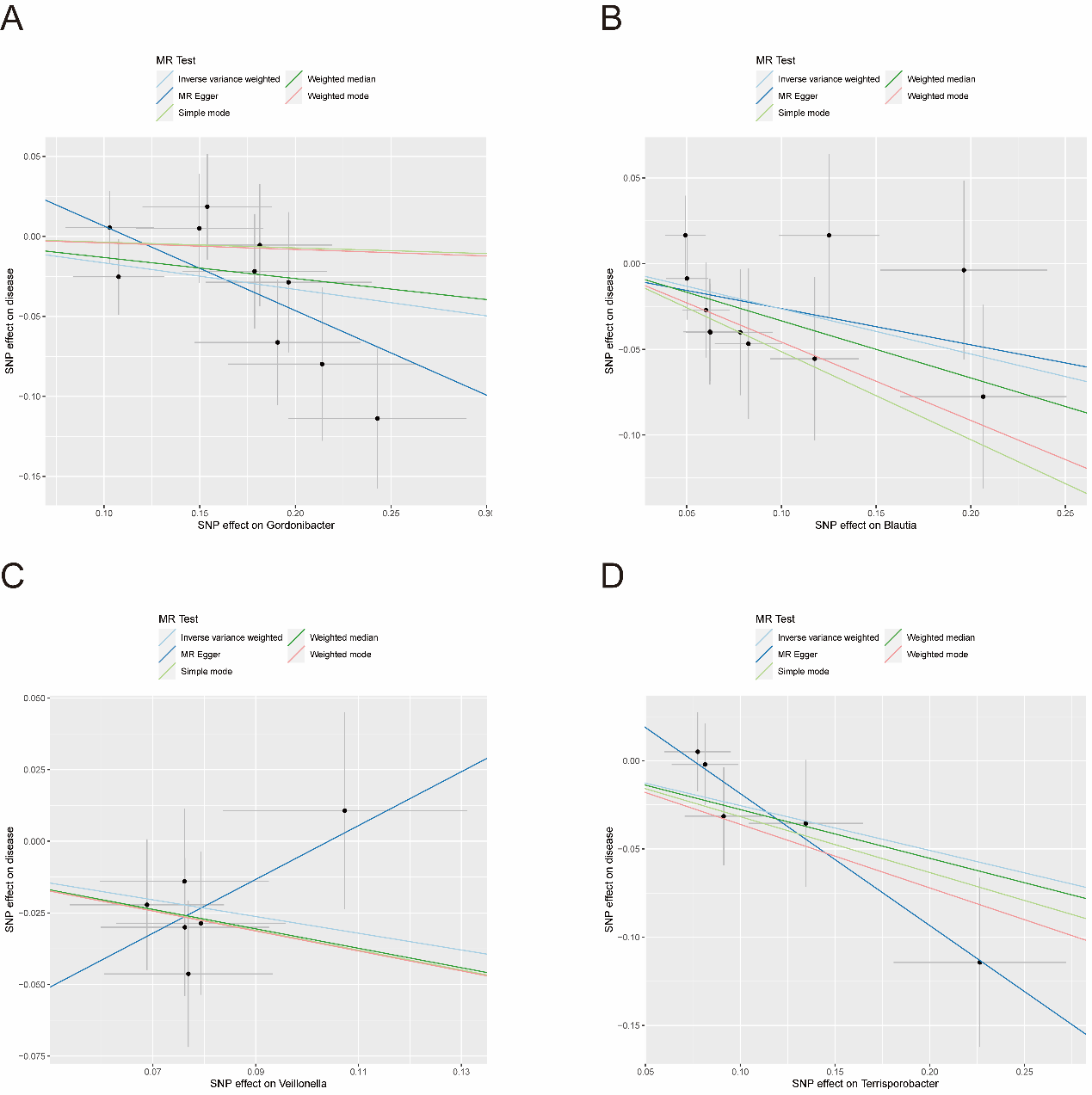


(A) genus Gordonibacter

(B) genus Blautia

(C) genus Veillonella

(D) genus Terrisporobacter

**Figure S9.** Scatter plots for the effect of Gut microbiota on H1N1.


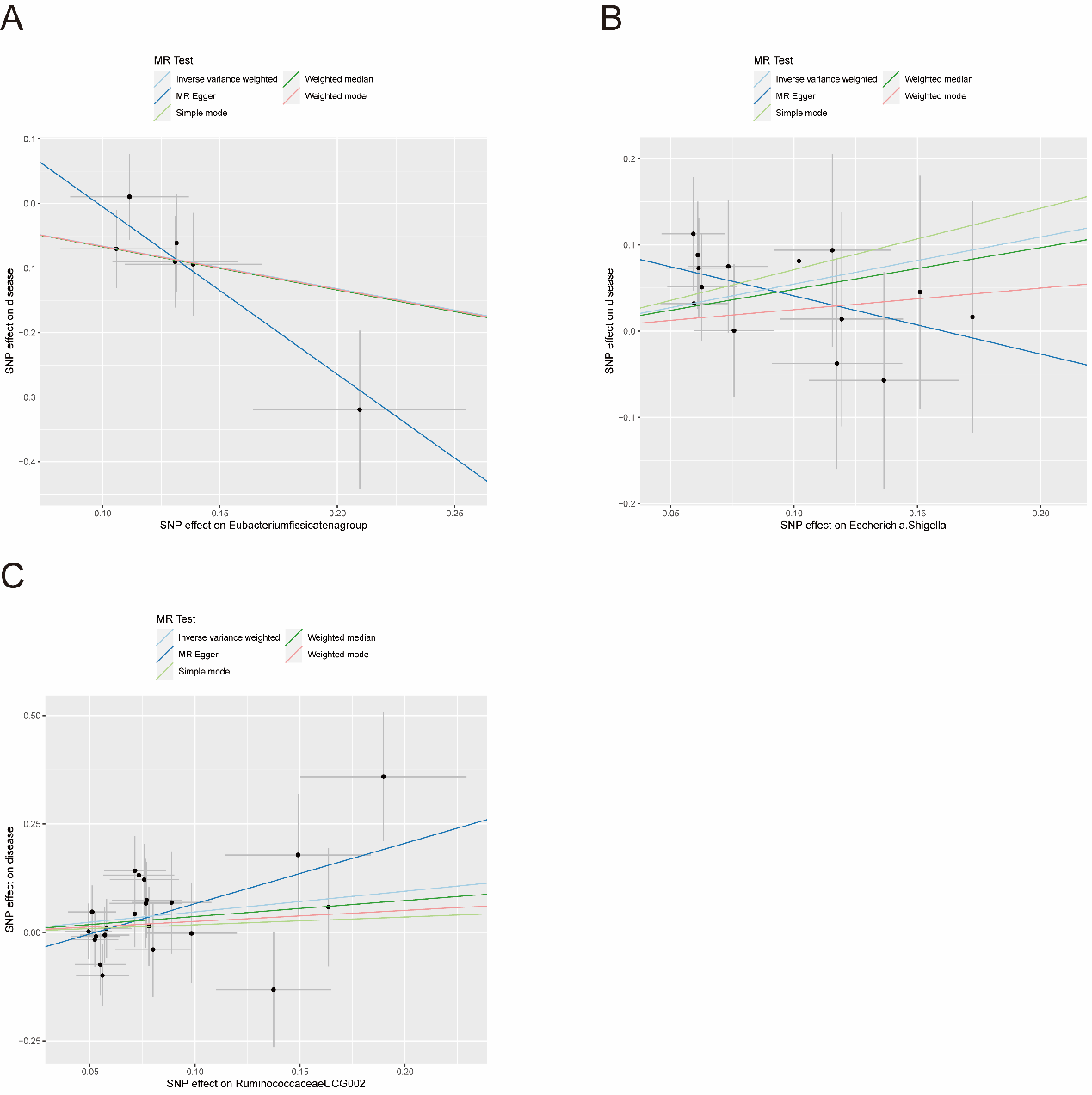


(A) genus Eubacteriumfissicatenagroup

(B) genus Escherichia.Shigella

(C) genus RuminococcaceaeUCG002

**Figure S10.** Scatter plots for the effect of Gut microbiota on HSV-1.


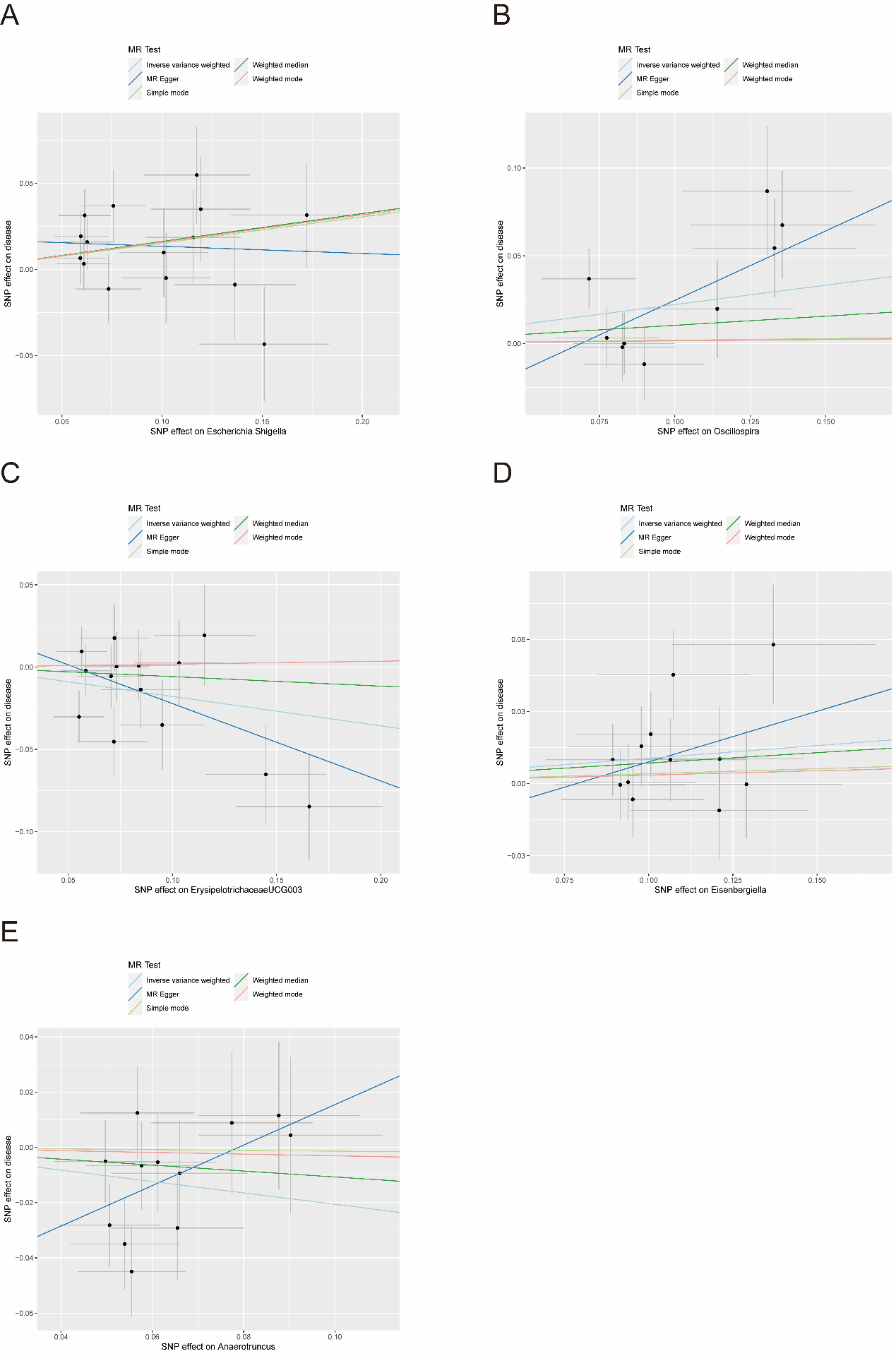


(A) genus Escherichia.Shigella

(B) genus Oscillospira

(C) genus ErysipelotrichaceaeUCG003

(D) genus Eisenbergiella

(E) genus Anaerotruncus

**Figure S11.** Forest plots for the effect of Gut microbiota on AdV.


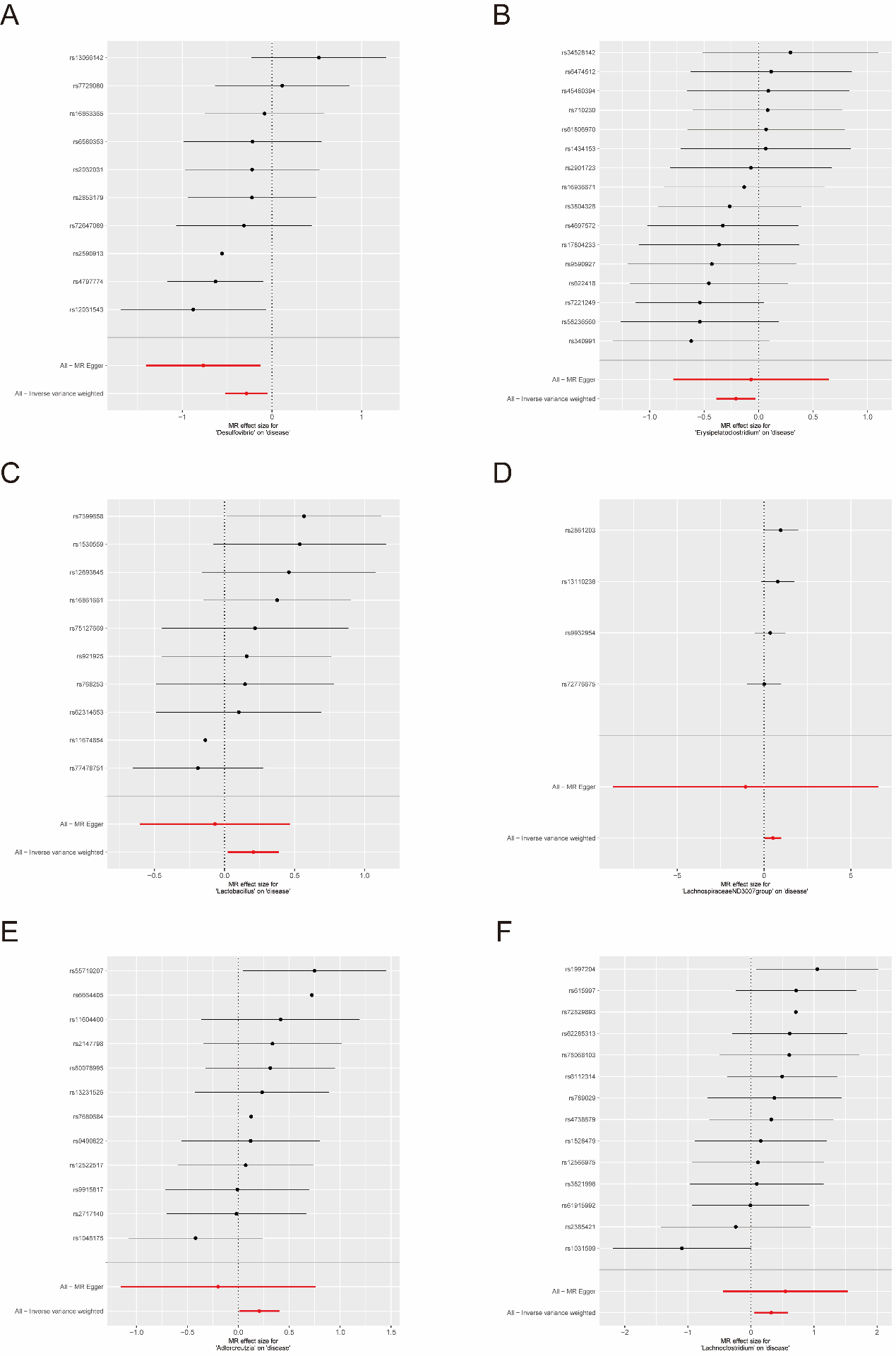


(A)MR effect size for "genus Desulfovibrio" on "AdV"

(B) MR effect size for "genus Erysipelatoclostridium" on "AdV"

(C) MR effect size for "genus Lactobacillus" on "AdV"

(D) MR effect size for "genus LachnospiraceaeND3007group" on "AdV"

(E) MR effect size for "genus Adlercreutzia" on "AdV"

(F) MR effect size for "genus Lachnoclostridium" on "AdV"

**Figure S12.** Forest plots for the effect of Gut microbiota on CMV.


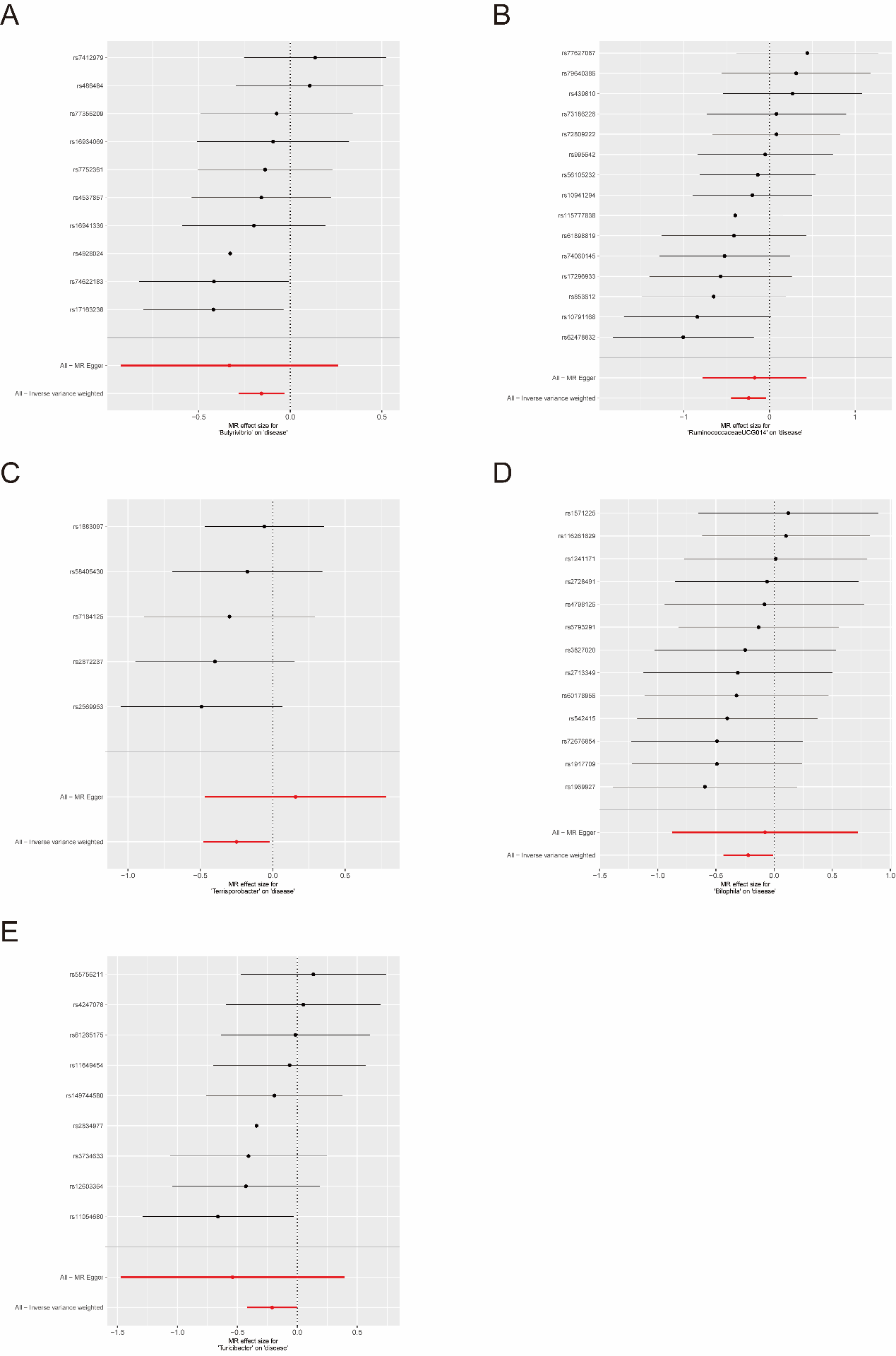


(A) MR effect size for "genus Butyrivibrio" on "CMV"

(B) MR effect size for "genus RuminococcaceaeUCG014" on "CMV"

(C) MR effect size for "genus Terrisporobacter" on "CMV"

(D) MR effect size for "genus Bilophila" on "CMV"

(E) MR effect size for "genus Turicibacter" on "CMV"

**Figure S13.** Forest plots for the effect of Gut microbiota on EBV.


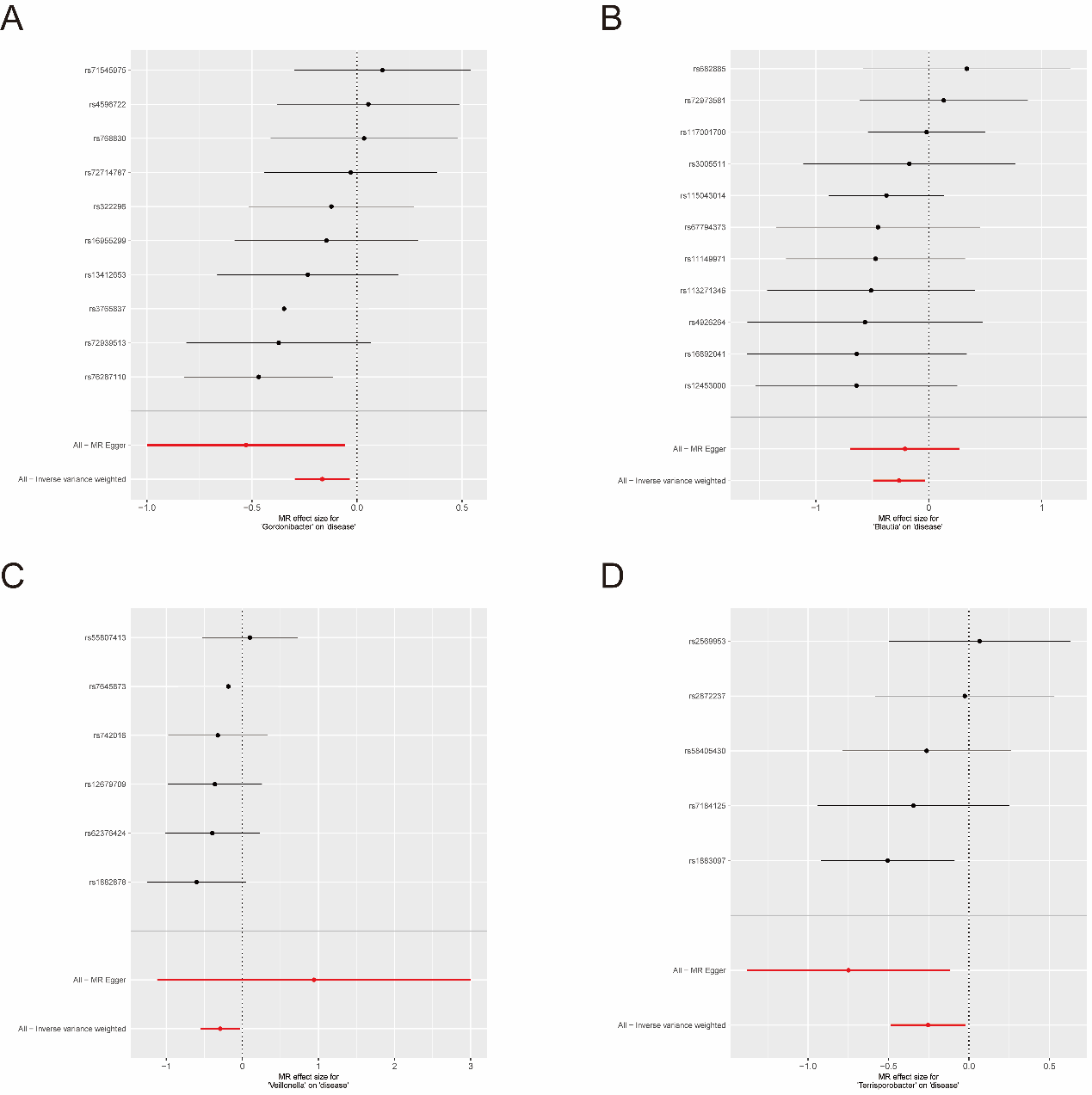


(A) MR effect size for "genus Gordonibacter" on "EBV"

(B) MR effect size for "genus Blautia" on "EBV"

(C) MR effect size for "genus Veillonella" on "EBV"

(D) MR effect size for "genus Terrisporobacter" on "EBV"

**Figure S14.** Forest plots for the effect of Gut microbiota on H1N1.


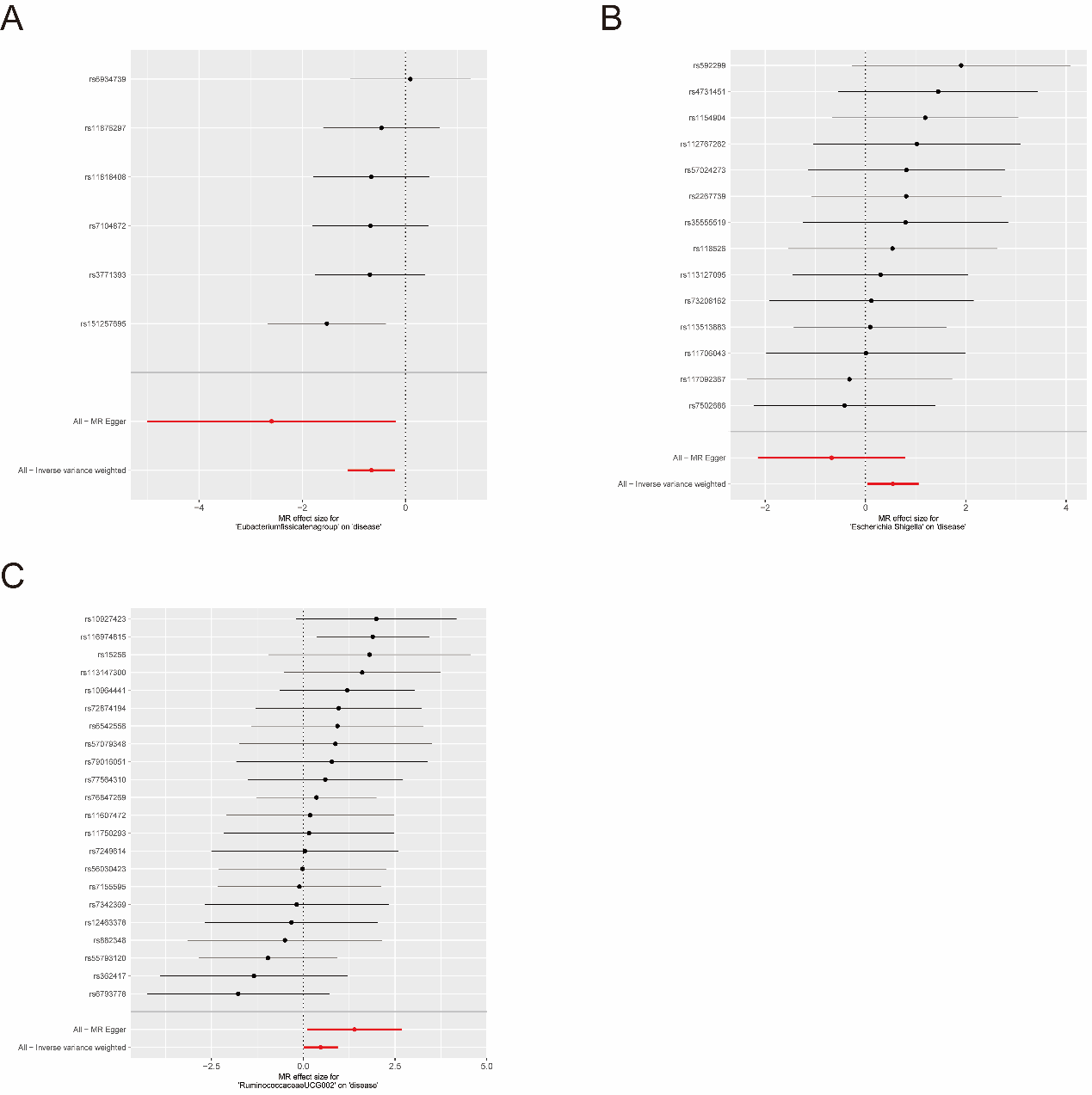


(A) MR effect size for "genus Eubacteriumfissicatenagroup" on "H1N1"

(B) MR effect size for "genus Escherichia.Shigella" H1N1"

(C) MR effect size for "genus RuminococcaceaeUCG002" on "H1N1"

**Figure S15.** Forest plots for the effect of Gut microbiota on HSV-1.


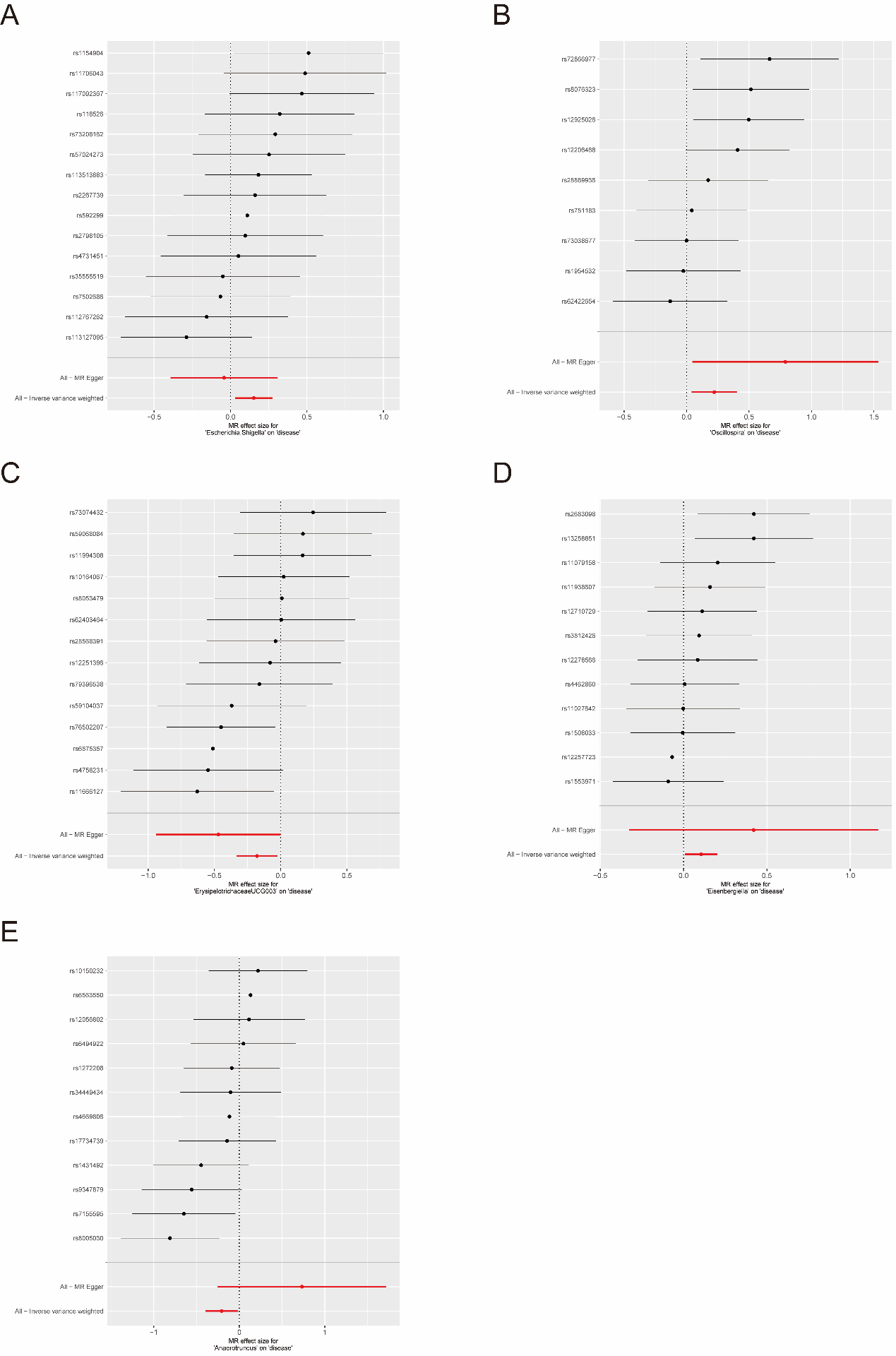


(A) MR effect size for "genus Escherichia.Shigella" on " HSV-1"

(B) MR effect size for "genus Oscillospira" on "HSV-1"

(C) MR effect size for "genus ErysipelotrichaceaeUCG003" on " HSV-1"

(D) MR effect size for "genus Eisenbergiella" on " HSV-1"

(E) MR effect size for "genus Anaerotruncus" on " HSV-1"
